# Effects of Resistance and Endurance Training Alone or Combined on Hormonal Adaptations and Cytokines in Healthy Children and Adolescents: A Systematic Review and Meta-analysis

**DOI:** 10.1186/s40798-022-00471-6

**Published:** 2022-06-21

**Authors:** Daniel Jansson, Ann-Sofie Lindberg, Elena Lundberg, Magnus Domellöf, Apostolos Theos

**Affiliations:** 1 Department of Community Medicine and Rehabilitation, Section of Sports Medicine, Umeå University, Linnaeus väg 9, 901 87 Umeå, Sweden; 2 Department of Clinical Sciences, Pediatrics, Umeå University, Umeå, Sweden; 3 Winternet, Boden, Sweden; 4 Umeå School of Sport Sciences, Umeå University, Umeå, Sweden

**Keywords:** Testosterone, GH, IGF-I, Cortisol, IL-6, TNF-α, SHBG, Pediatric

## Abstract

**Background:**

No previous systematic review has quantitatively compared the effects of resistance training, endurance training, or concurrent training on hormonal adaptations in children and adolescents. Objective was to examine the effects of exercise training and training type on hormonal adaptations in children and adolescents.

**Methods:**

A systematic literature search was conducted in the following databases: PubMed, Web of Science, and EBSCO. Eligibility criteria were: population: healthy youth population sample (mean age < 18 years); intervention: resistance training, endurance training, or concurrent training (> 4 weeks duration); comparison: control group; outcome: pre- and post-levels of hormones and cytokines; and study design: randomized and non-randomized controlled trials. We used a random-effect model for the meta-analysis. The raw mean difference in hormones from baseline to post-intervention was presented alongside 95% confidence intervals (CI). Further, the certainty of evidence quality and the risk of bias were assessed.

**Results:**

A total of 3689 records were identified, of which 14 studies were eligible for inclusion. Most studies examined adolescents with fewer studies on children (age < 12 years, *N* = 5 studies) and females (*N* = 2 studies). Nine exercise training programs used endurance training, five studies used resistance training, and no eligible study used concurrent training. The meta-analysis showed no significant effect of exercise training on testosterone (MD = 0.84 nmol/L), cortisol (MD = − 17.4 nmol/L), or SHBG (MD = − 5.58 nmol/L). Subgroup analysis showed that resistance training significantly increased testosterone levels after training (MD = 3.42 nmol/L) which was not observed after endurance training (MD = − 0.01 nmol/L). No other outcome differed between training types. Exercise training resulted in small and non-significant changes in GH (MD = 0.48 ng/mL, *p* = 0.06) and IGF-I (MD = − 22.90 ng/mL, *p* = 0.07). GH response to endurance training may be age-dependent and evident in adolescents (MD = 0.59 ng/mL, *p* = 0.04) but not when children and adolescents are pooled (MD = 0.48 ng/mL, *p* = 0.06). Limited evidence exists to conclude on IL-6 and TNF-α effects of exercise training. Assessments of GRADE domains (risk of bias, consistency, directness, or precision of the findings) revealed serious weaknesses with most of the included outcomes (hormones and cytokines).

**Conclusions:**

This systematic review suggests that exercise training has small effects on hormonal concentrations in children and adolescents. Changes in testosterone concentrations with training are evident after resistance training but not endurance training. GH's response to training may be affected by maturation and evident in adolescents but not children. Further high-quality, robust training studies on the effect of resistance training, endurance training, and concurrent training are warranted to compare their training-specific effects.

*Registration*: PROSPERO: CRD42021241130.

## Key Points

- The main findings of this systematic review and meta-analysis suggest that exercise training has a small effect on hormonal concentrations in healthy children and adolescents (< 18 years).
- Basal levels of GH significantly increased after endurance training in adolescents, but this effect was not significant when studies on adolescents and children were pooled. At least for GH, hormonal adaptations to exercise training may be affected by maturation. None of the other outcomes were affected by age.
- Training type may affect hormonal adaptations to exercise training. Resistance training induced a higher increase in testosterone concentration compared to endurance training. No effect of training mode was evident for concentrations of SHBG or cortisol. All studies examining GH and IGF-I used endurance training, and subgroup analysis was therefore not conducted.
- Conflicting evidence exists on the effects of exercise training on IL-6 and TNF-α.

## Background

Different training types may have different effects on muscular strength, cardiorespiratory fitness, and other health outcomes in children and adolescents [1, 2]. Regular exercise promotes the development of musculoskeletal, cardiovascular, respiratory systems and affects metabolism [3]. However, in children and adolescents, these effects interact with the endocrine system, which affects growth, metabolism, pubertal, and neuropsychological development [4]. It has been shown that physical activity affects the endocrine system during acute and prolonged exercise in adults [5, 6], children and adolescents [7]. Traditionally, hormones like growth hormone (GH), insulin-like growth factor 1 (IGF-I), and sex steroids, mainly testosterone, have been studied due to their role in tissue growth and muscle hypertrophy [5].

### Hormonal and Cytokine Adaptations to Physical Training

Hormonal adaptations associated with exercise training differ in children and adolescents compared to adults, possibly due to differing stages of maturation [8]. The onset of puberty corresponds to rapid physiological changes in the secretion of sex steroids and GH-IGF-axis hormones [3, 9–11]. During this period, the effects of exercise training on anabolic hormones and catabolic/inflammatory mediators are believed to be particularly important [3, 12]. Specifically, males gain greater muscular strength during puberty compared to females as a result of a significant increase in their testosterone levels, while there are no sex differences in muscular strength before puberty [13]. In accordance, gains in muscular strength after resistance training among prepubertal children should be attributed to neural rather than morphological muscle adaptations [14–16]. However, there are limited data on hormonal [7] and morphological adaptations [8] to exercise training in children and adolescents. Some suggest that morphological adaptations occur in children but are possibly more subtle in children than adults [17–19].

In pubertal males, exercise training can cause acute responses in hormones like testosterone and cortisol, induce chronic changes [3], and may even influence growth and maturation. Both testosterone and cortisol levels tend to increase following acute resistance training in adults, but children and adolescents' response is less clear [7]. Some evidence suggests that exercise-induced acute cortisol and testosterone responses to resistance training may depend on maturation [20].

More recently, the focus has shifted toward examining the GH-IGF-I axis in pediatric exercise physiology [21–24]. The GH-IGF-I axis is a system of growth mediators (IGFs, IGF-binding proteins, and IGF receptors) that has an essential role in normal growth, development, and cellular differentiation [25, 26]. The link between exercise training and the GH-IGF-I axis has been reported in cross-sectional studies, with a higher GH secretion and IGF-I concentration in fitter adults [27, 28] and adolescents compared to less trained participants [29, 30]. In addition, both GH and IGF-I increase significantly as a result of endurance training stimuli [28, 31–39]. The acute GH response to an endurance-type training session also seems to depend on maturation [5, 32, 33, 36, 40, 41], with a lower response in prepubertal compared to pubertal children. The acute response to exercise in IGF-I concentration is less studied in children and adolescents but hypothesized to increase [12]. However, most studies have failed to find a significant increase in IGF-I concentrations after training [38, 41]. Even though IGF-I is the downstream hormone stimulated by GH, some have suggested that IGF-I is not necessarily dependent on GH since IGF-I peaks earlier than GH after exercise [38, 42].

Short-term (5 weeks) endurance-type training programs in children have resulted in a catabolic rather than an expected anabolic activation of the GH-IGF-I axis [22]. The inhibition of the GH-IGF-I-axis has been suggested to be caused by simultaneous activation of catabolic proinflammatory cytokines such as interleukin 6 (IL-6) and tumor necrosis factor-α (TNF-α) [22, 43]. Previous studies have found that intense acute bouts of sport-specific training sessions increase the inflammatory cytokines in male [22] and female adolescents [23]. It has been suggested that levels of proinflammatory cytokines will fall back to normal values after a longer training period that improves physical performance and thereby suppression of IGF-I diminishes [44]. A successful training adaptation may decrease proinflammatory cytokines and rebound anabolic activation of the GH-IGF-I axis, causing IGF-I to increase above pretraining levels [44]. The exact role of the inflammatory cytokine activity in growth and development is still unclear and how it changes with long-term training.

It is well documented that children, and adolescents can increase cardiorespiratory endurance and muscular strength [2]. Less clear is how children and adolescents, hormonal systems adapt to long-term training [3, 7]. A systematic review and meta-analysis showed that endurance training effectively reduced fasting insulin levels in obese children and adolescents more than resistance training or endurance training [45]. However, obesity in children has been demonstrated to decrease GH and insulin response to exercise, thus affecting the hormonal responses compared to lean children [37, 38, 46]. According to our knowledge, no previous systematic reviews and meta-analyses have investigated whether training types induce different long-term effects on other hormonal outcomes in a healthy population. Knowledge of which physiological mechanisms underpin children’s response to training is essential to designing safe and effective training programs. Therefore, this systematic review and meta-analysis aimed to determine the effects of exercise training on hormonal adaptations and cytokines in healthy children and adolescents. Further, we reviewed and compared training types and their effects on hormones and cytokines in children and adolescents.

## Methods

The systematic review and meta-analysis were performed in accordance with the Preferred Reporting Items for Systematic Reviews and Meta-Analysis (PRISMA) statement [47, 48]. The protocol was specified in advance and registered in PROSPERO international prospective register of systematic reviews (CRD42021241130).

### Search Strategy

The literature search was performed on the April 15, 2021, and updated February 23, 2022, in PubMed, Web of Science (core collection), and EBSCO (Academic Search Premier, CINAHL, MEDLINE, SPORTDiscus). The search strategy (Table 1) included the following Boolean search syntax for training mode and hormonal response (youth OR children OR adolescents OR adolescence OR teens OR teenager OR boys OR girls OR child OR young OR junior) AND (hormones OR endocrine OR hormonal OR cytokine) AND (exercise OR training) AND (aerobic OR endurance OR interval OR strength OR resistance OR concurrent OR combined). The following database filters were applied when available: participant age (child: birth—18 years) and language (English).

**Table 1:** Search strategy used for each database

Database	Search words
PubMed	*
Web of Science	**
EBSCO	***

^*^All fields: ("Adolescent"[Mesh] OR "Child"[Mesh] OR youth OR child* OR adolescent* OR adolescence OR teen* OR teenager OR boy* OR girl* OR young OR junior) AND ("Hormones"[Mesh] OR hormone* OR endocrine OR hormonal OR "Cytokines"[Mesh] OR cytokine*) AND ("Exercise"[Mesh] OR training) AND ("physical endurance"[Mesh] OR "resistance training"[Mesh] OR aerobic OR endurance OR interval OR strength OR resistance OR concurrent OR combined)

^**^TOPIC: (youth OR children OR adolescents OR adolescence OR teens OR teenager OR boys OR girls OR child OR young OR junior) AND TOPIC: (hormones OR endocrine OR hormonal R cytokine) AND TOPIC: (exercise OR training) AND TOPIC: (aerobic OR endurance OR interval OR strength OR resistance OR concurrent OR combined)

^***^All fields: (youth OR children OR adolescents OR adolescence OR teens OR teenager OR boys OR girls OR child OR young OR junior) AND (hormones OR endocrine OR hormonal OR cytokine) AND (exercise OR training) AND (aerobic OR endurance OR interval OR strength OR resistance OR concurrent OR combined)

### Eligibility Criteria

The study criteria were formed according to the PICOS (population (P), intervention (I), comparator (C), outcomes (O), and study design (S)) guidelines [48, 49]. Participants: healthy children and adolescents (mean age ≤ 18 years) were included in the analysis. Studies on overweight or obese populations (BMI for age > 85th percentile) were excluded because their hormonal response to exercise differs compared to lean participants [37, 38, 46]. Overweight or obese was defined as stated in the original studies, typically with a BMI for age above 85th percentile. Studies with both children and adults were included if results were reported separately for the child group. Intervention: resistance training, endurance training, or concurrent training with a duration of at least four weeks were included. Comparator: only studies with at least one control group were included. Outcome: the study contained data on at least one proinflammatory cytokine: TNF-α or IL-6 or at least one of the following hormones: GH, testosterone, IGF-I, SHBG, or cortisol measured before and after a period of training. Only studies examining changes in hormonal or cytokine concentrations pre- to post-training, termed chronic changes (> 4 weeks of training), were considered. Study design: both randomized or non-randomized controlled trials were included. Further, only studies published in English and available in full-text were included.

### Study Selection

After removing duplicates, one review author (DJ) screened the titles and dismissed irrelevant articles. The screening process strictly adhered to the a priori eligibility criteria published in the PROSPERO protocol. Two independent authors (DJ and AT/AL) screened the abstracts, and articles that did not meet the eligibility criteria were excluded. At this stage, only the studies that clearly did not match the eligibility criteria were excluded (e.g., populations such as adults and individuals with chronic diseases; study design such as cross-sectional studies and acute studies; and types of reports such as conference papers, and reviews). All potential articles advanced to the next step of the screening process and were carefully examined in full-text by two independent authors (DJ and AT/AL) and included only if they met the inclusion criteria described above. Any disagreement was resolved by discussing and revisiting the original paper. After identifying relevant articles, we manually searched their reference lists to increase sensitivity.

### Data Extraction

We developed a standardized digital data extraction form (based on Cochrane Consumers and Communication Review Groups’ data extraction form [50]). The following information was extracted: (1) study design; (2) characteristics of participants (e.g., sex, body mass, body height, biological and chronological age); (3) study aim; (4) intervention characteristics (e.g., training type, intensity, duration, frequency); and (5) hormonal and cytokine outcome (e.g., pre- and post-values, blood or saliva). Two review authors independently extracted the data from the articles. Any inconsistencies in data extraction were resolved by discussion and revisiting the original paper. The original study authors were contacted for clarification where critical data were missing or not reported fully. If no answer was obtained within two weeks, a reminder was sent to the corresponding author and the co-authors. If the authors did not answer our e-mails, the study was left out of the quantitative analyses. Data presented only in figures were extracted using a validated [51] WebPlotDigitizer software, version 4.5 (https://www.automeris.io/WebPlotDigitizer/).

### Risk of Bias Assessment

Two review authors (DJ and AT/AL) independently assessed the risk of bias using the Cochrane Collaboration’s Tool for Assessing Risk of Bias [50]. The two data sets were cross-referenced for any discrepancies. A third reviewer settled disagreements.

### GRADE of Evidence

The Grading of Assessment, Development and Evaluation (GRADE) criteria were used to interpret the findings and summarize the level of evidence [52, 53]. Each outcome in the study was evaluated according to the GRADE procedure. Evidence of findings was downgraded from “high certainty” by one level for serious concerns (two levels for very serious) for each criteria: risk of bias, indirectness of evidence, inconsistency of findings, imprecision of effect estimates, or publication bias across studies. Three review authors (DJ, AT, and AL) independently assessed each outcome according to the GRADE procedure, and any discrepancies and disagreements were settled by discussion.

### Statistical Analysis

A meta-analysis was completed using the Review Manager version 5.4.1 (Copenhagen: The Nordic Cochrane Centre, Cochrane) software. A random-effect model was used for the meta-analysis to calculate weighted mean differences (MD) in hormonal outcome from baseline to post-intervention between the groups. Only outcomes for which data were available from three or more studies were included in the meta-analyses and otherwise briefly described in the text. A primary meta-analysis was performed with all training studies included per outcome (Exercise training). The subgroup analysis was carried out considering the training modalities (resistance, endurance, or concurrent) used in the studies. Two studies included two or more intervention groups and one comparator group [54, 55], and results were reported separately since they examined different biological (e.g., prepubertal vs. pubertal) or chronological (11 vs. 15 years old) age groups. One study group was reported in two separate studies [55, 56], and therefore, only one of the groups was included in the meta-analysis [56].

Statistical heterogeneity in the systematic review and meta-analysis was assessed using Q and I^2^ statistics [57]. Thresholds for I^2^ were interpreted according to Cochrane [57]: 0–40% might not be important. 30–60% may represent moderate heterogeneity, 50–90% may represent substantial heterogeneity, and 75–100% considerable heterogeneity. In addition, heterogeneity was presented visually in forest plots with 95% confidence intervals. We calculated the pooled mean difference between exercise training and control groups for the absolute change in hormonal levels with 95% confidence intervals (CI). When different measures of variability were presented (e.g., standard error) in the original article, we converted them to SD following section 6.5.2.3 of the Cochrane Handbook [58]. The robustness of the meta-analysis was evaluated using sensitivity analysis for biological age, following the guidelines in section 2.9.97 in the Cochrane Handbook [58]. The level of statistical significance was set at *α* = 0.05.

## Results

### Description of Studies

**Fig. 1:**
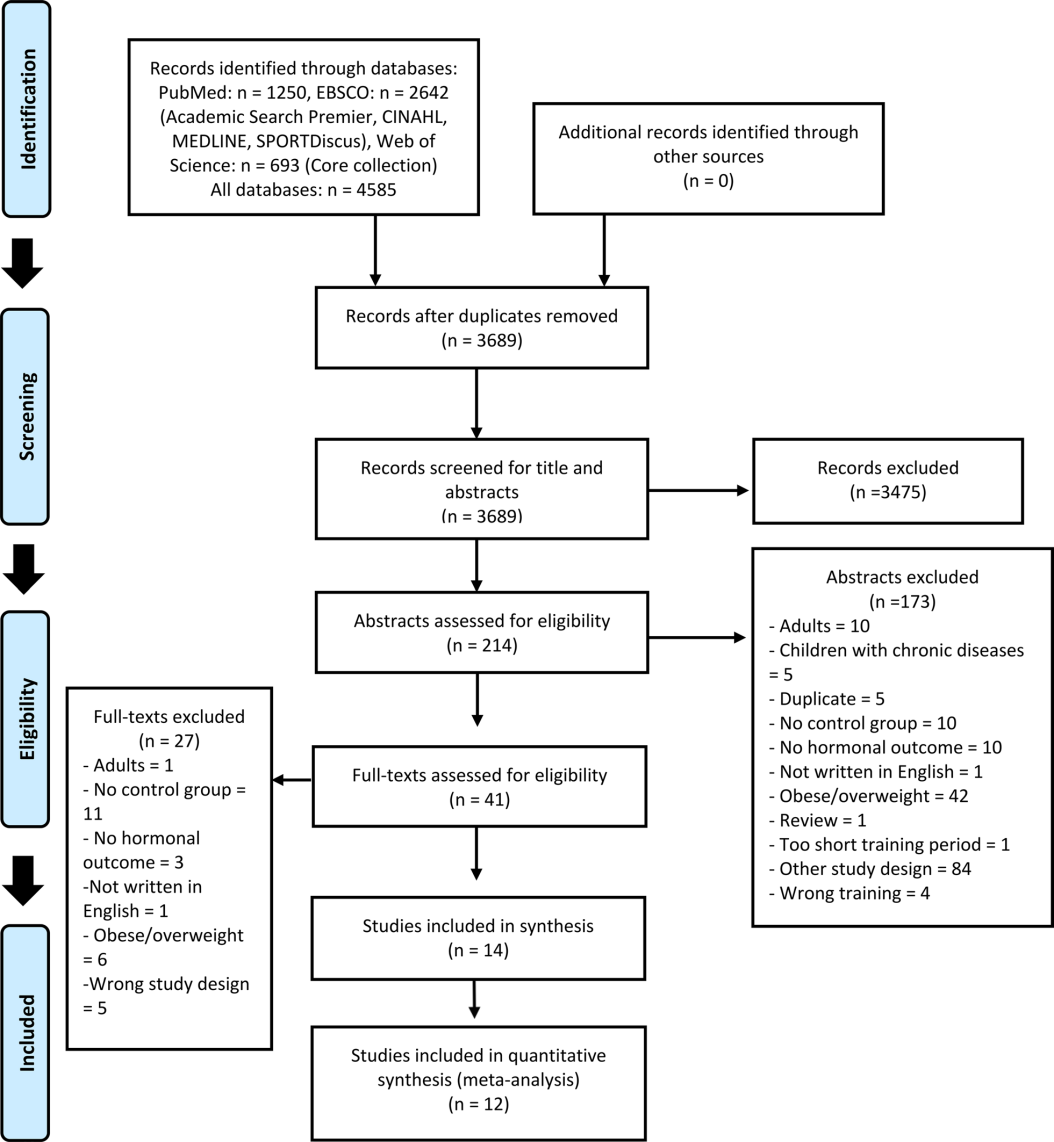
Flowchart for inclusion and exclusion of studies

The database search resulted in 3689 potential studies for inclusion after duplicates had been removed (Fig. 1). Screening of abstracts and titles resulted in 41 potentially relevant studies that were carefully assessed in full-text for eligibility. In total, 14 studies met the inclusion criteria, and 12 were included in the meta-analysis [29, 43, 54–56, 59–67]. Details of participant characteristics are presented in Table 2. The systematic review and meta-analysis included 445 participants (347 males and 98 females) with mean ages for study populations ranging between 9 and 17 years. Most of the studies examined adolescents (> 12 years old) [59–63, 65, 66] with fewer studies examining children (< 12 years old) [29, 43, 56, 64, 67] and two studies included both age groups [54, 55]. Ten studies examined untrained participants [29, 43, 54–56, 62–64, 66, 67], and four studies examined trained participants [59–61, 65]. Sample sizes ranged from 16 to 69 participants. All studies measured hormonal concentration in blood, except for one that measured in saliva [65]. Biological age was reported in the majority of the studies, however, not all [59, 60, 65–67]. Five of the included studies examined the effects of resistance training [55, 56, 59, 61, 65], nine studies examined endurance training [29, 43, 54, 60, 62–64, 66, 67], and no study was found examining the effects of concurrent training (Table 2). The mean training duration of the included studies was 8 weeks (range from 5 to 24 weeks) with a training frequency ranging from two to five training sessions per week.

**Table 2:** Participant and intervention characteristics for all included studies

Study	Group	*N*	Age (yrs)	Sex	Height (cm; mean ± SD)	Weight (kg; mean ± SD)	Population	Study duration (weeks)	Training frequency (sessions) (days/week)	Training description
Eliakim et al. [62]	ET	10	15–17	F	161.4 ± 1.8	61.6 ± 3.9	High-school students	5	5	Endurance-type training, consisting of running, aerobic dance, competitive sports (e.g., basketball)
	C	6	15–17	F	157.5 ± 1.5	52.9 ± 6.6				
Zakas et al. [54]	ET^a^	10	10	M	141.2 ± 6.3	38.6 ± 8.2	Untrained children and adolescents	12	3	Interval training; 50 min/session, alternating heavy (80–86% HR_max_) and light intervals (30–40% HR_max_) on cycle ergometer
	C^a^	8	10	M	138.3 ± 6.6	35.7 ± 6.0				
	ET^b^	10	13	M	159.7 ± 7.1	51.7 ± 9.1				
	C^b^	9	13	M	157.8 ± 4.9	48.8 ± 6.4				
	ET^c^	9	16	M	175.4 ± 4.9	72.4 ± 13.3				
	C^c^	9	16	M	173.0 ± 7.8	67.6 ± 12.0				
Büyükyazi et al. [60]	ET^a^	12	15.08 ± 0.4	M	175.4 ± 5.4	64.1 ± 7.1	Junior male basketball players	8	3	Continuous running: 60 min/session, distance: 4800 m, at 80% HR_max_
	ET^b^	12	15.07 ± 0.5	M	178.4 ± 6.2	66.0 ± 8.5				
	C	12	15.6 ± 0.5	M	174.1 ± 6.7	68.2 ± 8.1				
Eliakim et al. [63]	ET	20	16.0 ± 0.7^ m^	M	169.2 ± 1.6	61.0 ± 1.8	Untrained high-school students	5	5	Endurance-type training consisting of running, aerobic dance, competitive sports
	C	18		M	170.3 ± 1.6	66.2 ± 3.5				
Messinis et al. [64]	ET	9	10.33 ± 0.5	M	148.9 ± 5.4	41.9 ± 6.7	Untrained prepubertals	8	4	Continuous cycling: 45 min/session, at a intensity of 75% of PWC170 test result. Readjusted training volume halfway
	C	8	10.5 ± 0.5	M	149.9 ± 7.5	46.1 ± 9.8				
Scheett et al. [43]	ET	12	9.95 ± 0.4	M	140.9 ± 2.8	33.75 ± 2.7	Prepubertal and early pubertal students	5	5	Aerobic-type training: 90 min/session, consisted of age-appropriate and sport-specific drills and games and running, jumping, aerobic dance, and competitive sports (team sports and running games)
	C	14	10.04 ± 0.3	M	140.7 ± 1.9	36.83 ± 2.1				
Eliakim et al. [29]	ET	19	9.2 ± 0.1^ m^	F	134.7 ± 1.3 m	35.5 ± 2.3	Untrained prepubertals	5	5	Aerobic-type training: 2 × 45 min daily endurance-type training consisting of running, dancing, soccer
	C	20		F		32.2 ± 2.2				
Andrade et al. [67]	ET	14	11.7 ± 2.3	M + F	147.1 ± 3.9	40.6 ± 13.3	Children with moderate asthma	6	3	Continuous treadmill running: 20–30 min/session, 20 min/session at 70% of HR_max_ for week 1–2 and 30 min/session at 80% of HR_max_ for week 3–6
	C	17	11.4 ± 2.3	M + F	147.3 ± 6.1	45.2 ± 12.1				
Rosenbaum et al. [66]	ET	49	13.7 ± 0.1	M + F	164.3 ± 1.3	66.7 ± 3.2	High-school students	13	3	Endurance-type training (dance/non-contact kickboxing) or regular gym class
	C	20	13.6 ± 0.2	M + F	162.9 ± 1.9	64.6 ± 5.6				
Tsolakis et al. [56]	RT	9	11.78 ± 0.8	M	152.2 ± 5.9	43.0 ± 9.5	Untrained prepubertal	8	3	Upper-body resistance training, 60 min/session, using variable resistance machines, 6 exercises, 3 set × 10 reps at an intensity of 10RM
	C	10	12.0 ± 0.8	M	156.8 ± 8.68	43.2 ± 10.7				
Gorostiaga et al. [61]	RT	9	15.1 ± 0.7	M	173.1 ± 5.3	62.4 ± 7.1	Adolescent handball players	6	2	Heavy resistance training: 40 min/session, 6–3 reps × 4 sets of five exercises (bench press, squat, knee flexion curl, leg press and pecdec) at a variable resistance gym apparatus, at an intensity ranging from 50–90% of 1RM
	C	9	14.8 ± 0.4	M	170.6 ± 3.9	64.8 ± 13.7				
Gorostiaga et al. [59]	RT	8	17.3 ± 0.5	M	175.1 ± 5.4	66.8 ± 6.0	Adolescent soccer players	11	2	Periodized explosive strength training: lower body exercises, jump, and weight lifts, low-load and high-speed movements
	C	11	17.2 ± 0.7	M	177.4 ± 4.9	70.3 ± 6.7				
Tsolakis et al. [55]	RT^a^	9	11.78 ± 0.84	M	152.2 ± 5.91	43.0 ± 9.5	Untrained high-school prepubertal	8	2	Upper-body resistance training: 60 min/session, using variable resistance machines, 6 exercises, 10 reps × 3 set, at an intensity of 10RM
	C^a^	10	12.0 ± 0.82	M	156.8 ± 8.7	43.2 ± 10.7				
	RT^b^	13	14.92 ± 0.86	M	169.1 ± 9.3	55.8 ± 9.0				
	C^b^	10	14.9 ± 0.88	M	166.5 ± 5.0	56.8 ± 5.9				
Sarabia et al. [65]	RT	11	15.6 ± 0.7	M	170.9 ± 5.1 m	63.3 ± 9.1 m	Adolescent tennis players	6	2	Resistance training; exercises included supine bench press using free weights and parallel half squats in a smith- machine, squat jump (SJ) and countermovement jumps (CMJ), 60% 1RM
	C	9	15.25 ± 0.71	M						

Values are presented as mean ± SD

*RT* resistance training, *RT*^*a*^ resistance training group 1, *RT*^*b*^ resistance training group 2, *ET* endurance training, *ET*^*a*^ endurance training group 1, *ET*^*b*^ endurance training group 2, ETc endurance training group 3, *C* control group, *C*^*a*^ control group 1, *C*^*b*^ control group 2, *C*^*c*^ control group 3

^m^Mean value for all groups, including the control group

### Primary Analyses: Exercise Training

**Fig. 2:**
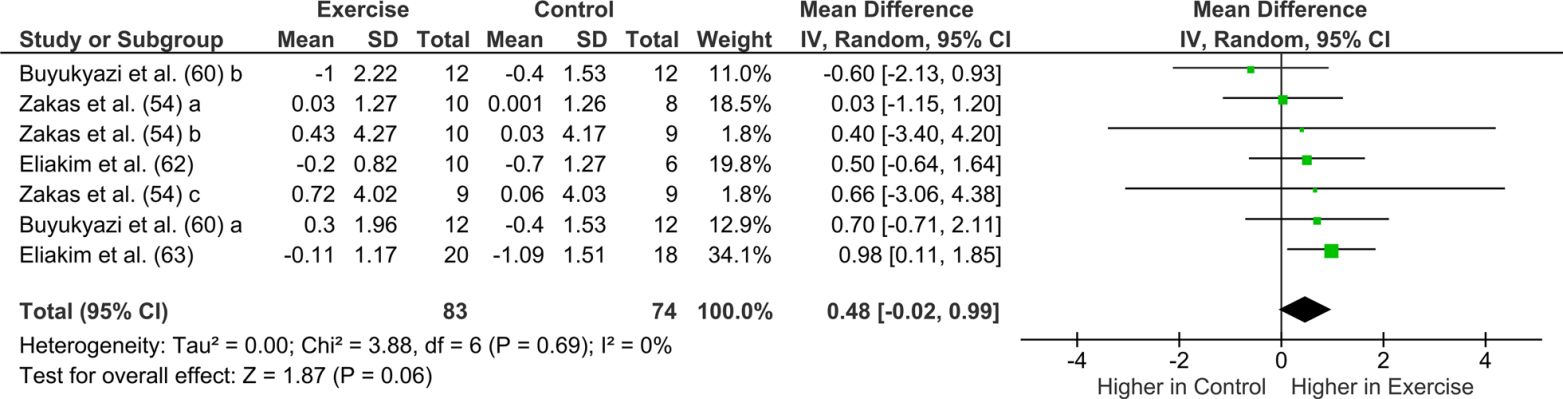
Forest plot of the effects of exercise training (only endurance training studies) compared with a control group on changes in growth hormone (GH). *CI* confidence intervals; *df* degrees of freedom; and *SD* standard deviation. A b and c refer to different study groups in the same publication

**Fig. 3:**
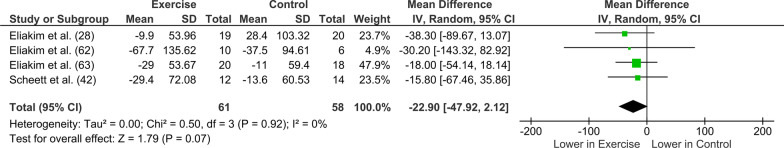
Forest plot of the effects of exercise training (only endurance training studies) compared with a control group on changes in insulin-like growth factor 1 (IGF-I). *CI* confidence intervals; *df* degrees of freedom; and *SD* standard deviation

**Fig. 4:**
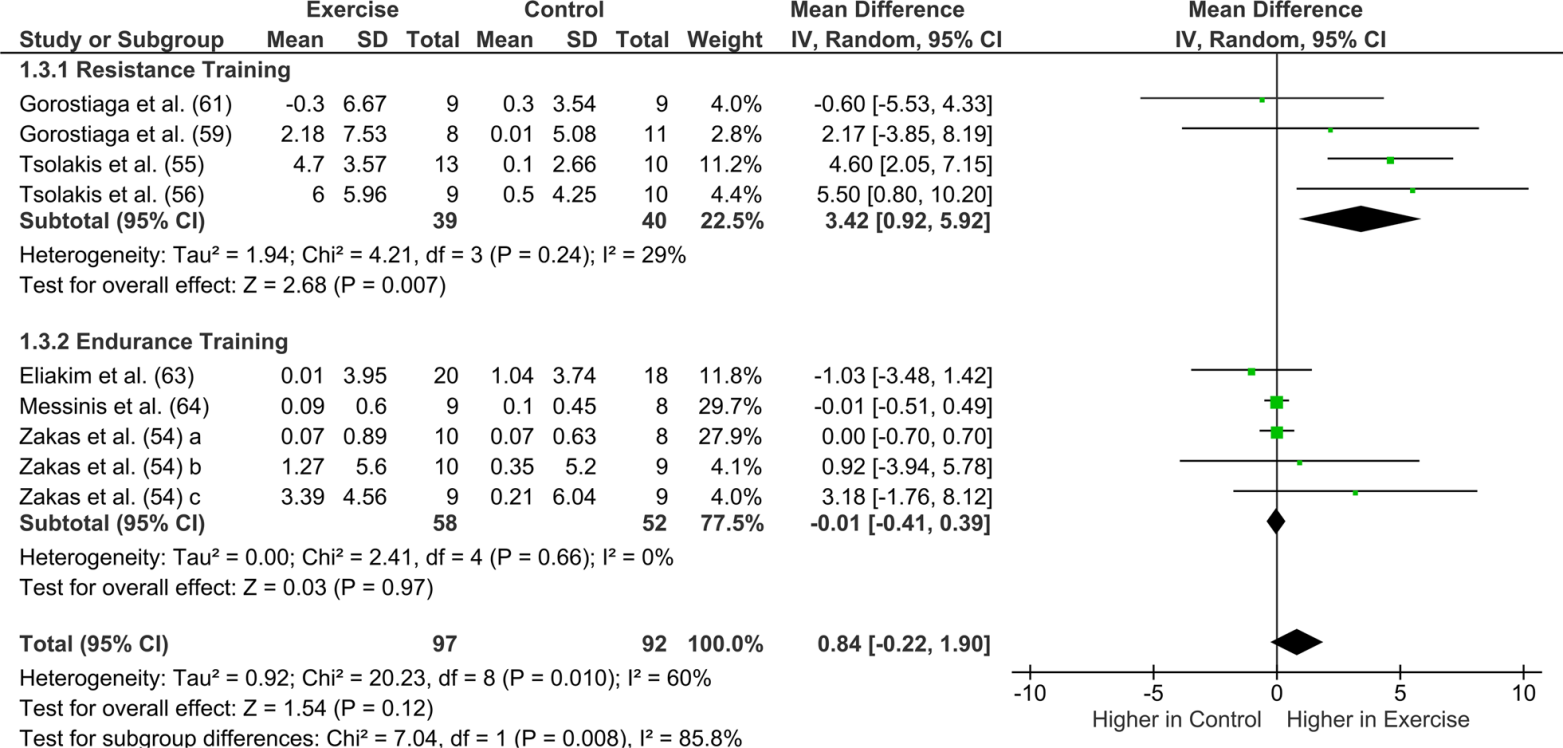
Forest plot of the effects of exercise training (pooled resistance and endurance training) vs. control group and resistance training vs. endurance training on changes in testosterone. *CI* confidence intervals; *df* degrees of freedom; and *SD* standard deviation. A b, and c refer to different study groups in the same publication

**Fig. 5:**
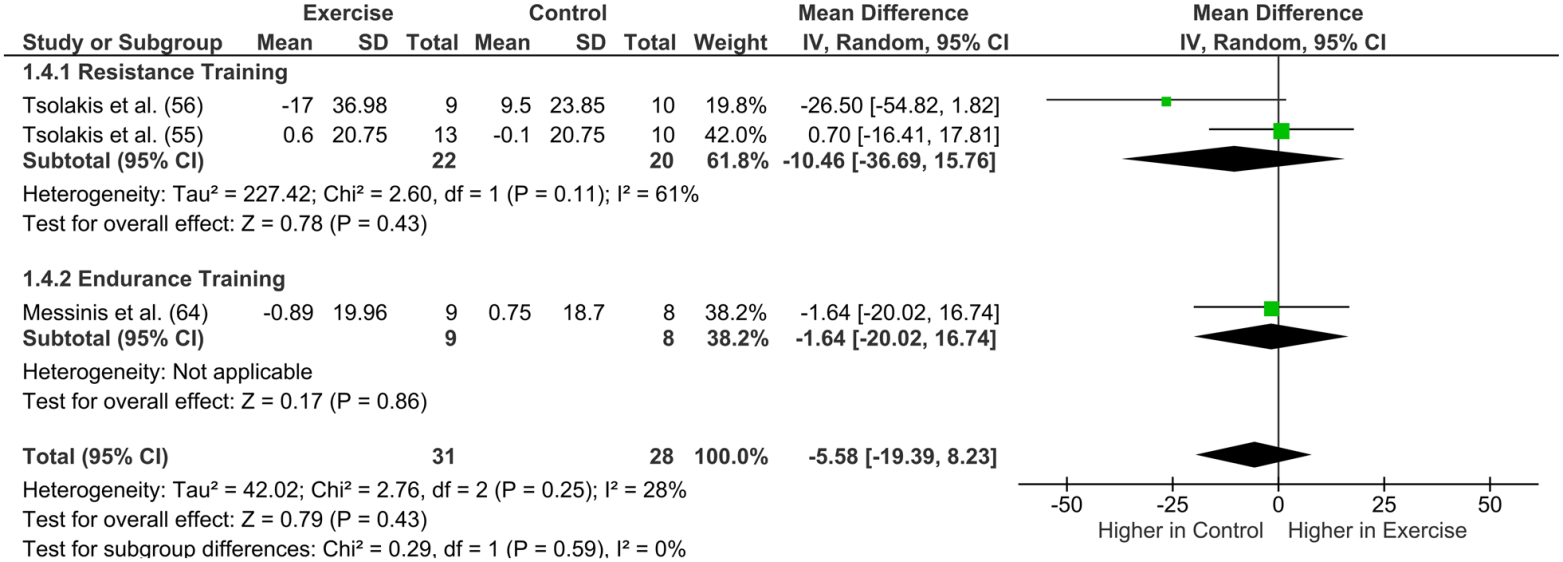
Forest plot of the effects of exercise training (pooled resistance and endurance training) vs. control group and resistance training vs. endurance training on sex hormone-binding globulin (SHBG). *CI* confidence intervals; *df* degrees of freedom; and *SD* standard deviation

**Fig. 6:**
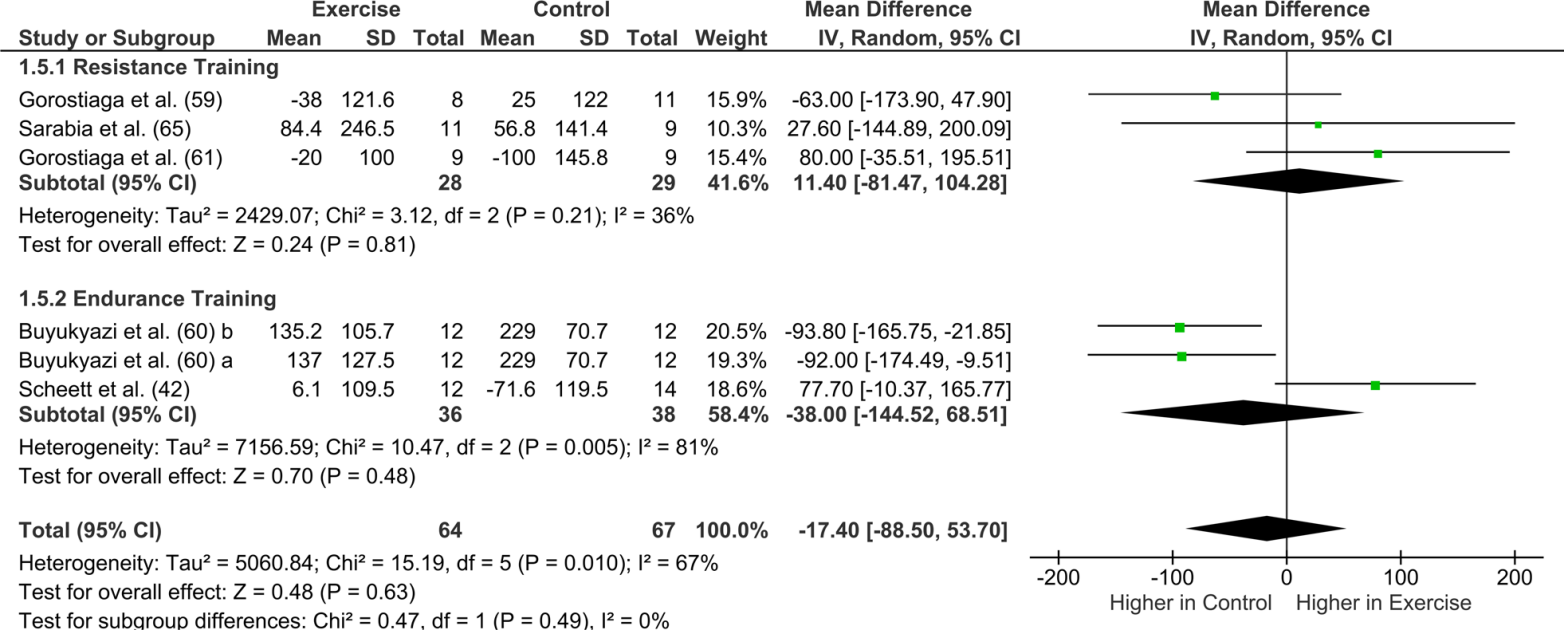
Forest plot of the effects of exercise training (pooled resistance and endurance training) vs. control group and resistance training vs. endurance training on changes in cortisol. *CI* confidence intervals; *df* degrees of freedom; and *SD* standard deviation. A and b refer to different study groups in the same publication

Twelve out of 14 studies included in the meta-analysis reported pre-and post-changes in hormones following exercise training. Testosterone (*n* = 7) followed by cortisol (*n* = 5) was the most studied hormone (Table 3). The meta-analysis showed that endurance exercise training was neither associated with a significant increase in GH pre- to post-training (mean difference: 048 ng/mL; [95% CI -0.02 to 0.99]; *I*^2^ = 0%, *Z* = 1.87, *p* = 0.06; Fig. 2) nor a significant decrease in IGF-I (mean difference: − 22.90 ng/mL [95% CI − 47.92 to 2.12], compared to a control condition (*Z* = 1.79 [*p* = 0.07]; Fig. 3). Changes in testosterone concentration (Fig. 4) pre- to post-training were not statistically significant (pooled exercise training; mean difference: 0.84 nmol/L [95% CI − 0.22 to 1.90], *Z* = 1.54 [*p* = 0.12]) compared to a control group. Between-study heterogeneity was moderate and significant (*I*^2^ = 60%, *p* = 0.010). Pooled exercise training was neither associated with any changes in SHBG concentration pre- to post-training (mean difference: − 5.58 nmol/L [95% CI − 19.39 to 8.23], *Z* = 0.79 [*p* < 0.43]; Fig. 5) nor a significant decrease in cortisol, compared to a control group (mean difference: − 17.40 nmol/L [95% CI − 88.50 to 53.70], *Z* = 0.48 [*p* = 0.63], Fig. 6).

**Table 3:** Summary of training studies per hormone (GH, IGF-I, testosterone, SHBG, and cortisol) reported separately for sex, age, and maturity

Hormone		Sex		Age		Maturity	
		Male	Female	Children (< 12 years)	Adolescents (> 12 years)	Prepubertals	Pubertals
GH	Total number of studies	3	1	1	3	1	3
	Resistance Training (studies)	0	0	0	0	0	0
	Endurance training (studies)	3	1	1	3	1	3
	Age (min–max)	10–16 year	15–17 year	10 year	13–17 year	10 year	13–17 year
	Maturity reported (studies)	2/3	1/1	1/1	2/3		
IGF-I	Total number of studies	2	2	2	2	2	2
	Resistance Training (studies)	0	0	0	0	0	0
	Endurance training (studies)	2	2	2	2	2	2
	Age (min–max)	9–16 year	9–17 year	9–11 year	15–17 year	9–11 year	15–17 year
	Maturity reported (studies)	2/2	2/2	2/2	2/2		
Testosterone	Total number of studies	7	0	4*	3	5*	3
	Resistance Training (studies)	4	0	2	2	2**	2
	Endurance training (studies)	3	0	2	2*	3	1
	Age (min–max)	10–17 year	0	11–12 year	13–17 year	9–11 year	12–17 year
	Maturity reported (studies)	6/7	0	4/4	2/3		
SHBG	Total number of studies	3	0	2	1	2	1
	Resistance Training (studies)	2	0	0	1	0	1
	Endurance training (studies)	1	0	2	0	2	0
	Age (min–max)	10–16 year	0	9–12 year	14–16 year	9–12 year	14–16 year
	Maturity reported (studies)	3/3	0	2/2	1/1		
Cortisol	Total number of studies	5	0	1	4	1	1
	Resistance Training (studies)	3	0	0	3	0	1
	Endurance training (studies)	2	0	0	1	1	0
	Age (min–max)	9–17 years	0	9–10 years	14–17 years	9–10 years	14–15 years
	Maturity reported (studies)	2/5	0	1/1	¼		

^*^ Zakas et al. [54] study included both a prepubertal group and a pubertal group. ** Tsolakis et al. prepubertal group is reported in two separate studies[55, 56]

**Fig. 7:**
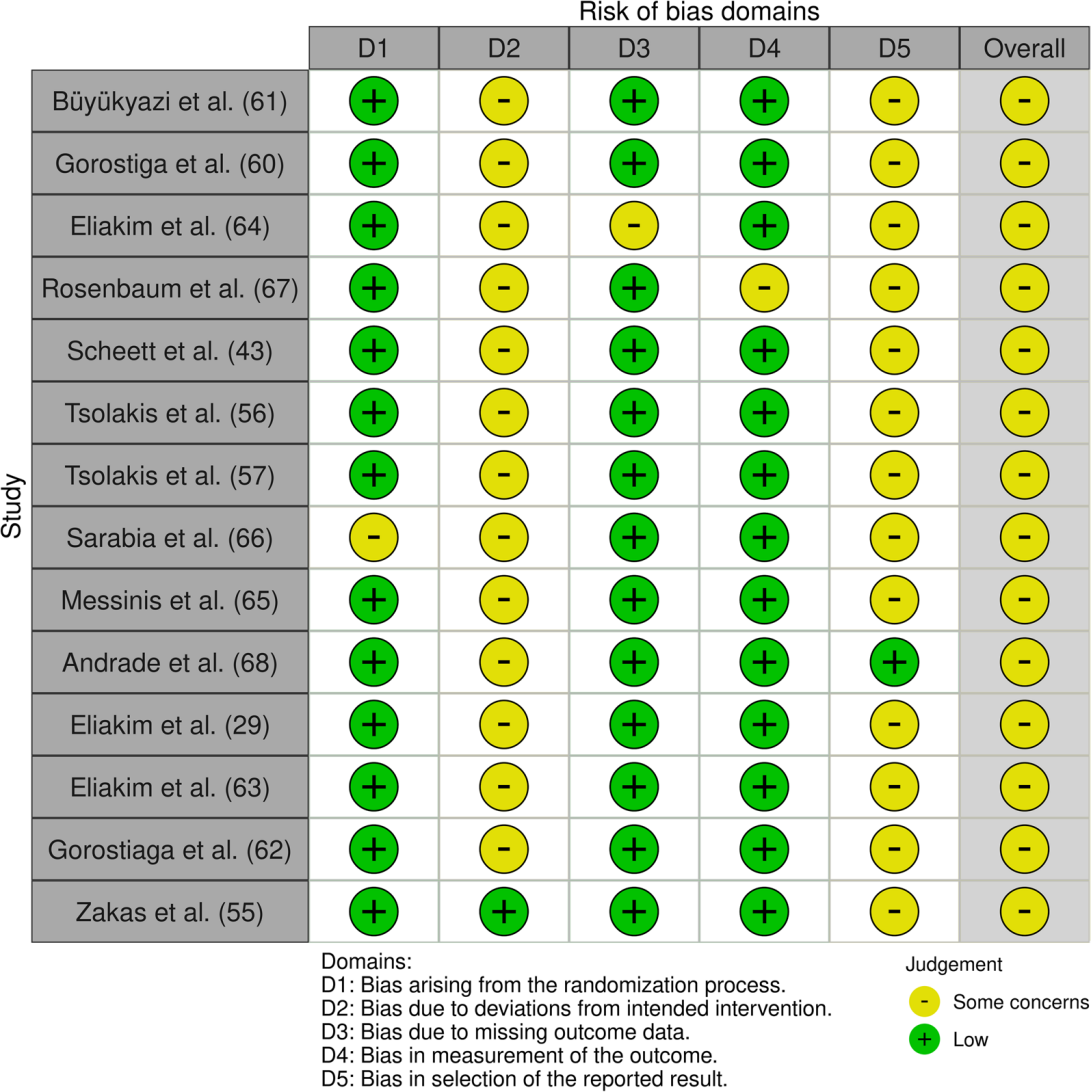
Risk of bias summary: review authors' assessment of each domain for all included studies (generated with the Review Manager Web, The Cochrane Collaboration, 2019)

The certainty of evidence (GRADE evaluation) was low to very low for all included hormonal outcomes (Table 4) because of concerns with the risk of bias (Fig. 7) and imprecise findings due to the low total sample size. In addition, some concerns were noted with SHBG and IGF-I because the same research group conducted the studies on SHBG (see [55, 56, 64]) and IGF-I (see [29, 30, 43, 63]).

**Table 4:** GRADE domains and overall certainty of evidence for the effects of exercise training on the outcomes

Outcome	Certainty assessment							No of patients		Effect		Certainty
	No of studies	Study design	Risk of bias	Inconsistency	Indirectness	Imprecision	Other considerations	Exercise training	No training	Absolute (95% CI)		
Growth hormone	4	Randomized trials	Serious ^a,b,c^	Not serious	Not serious	Serious ^d^	None	83	74	MD **0.48 ng/mL higher** (− 0.02 lower to 0.99 higher)		⨁⨁◯◯ LOW
IGF-I	4	Randomized trials	Serious ^a,b,c^	Not serious	Not serious	Serious ^d^	Publication bias strongly suspected ^e^	61	58	MD − **22.9 ng/mL lower** (− 47.9 lower to 2.1 higher)		⨁◯◯◯ VERY LOW
Testosterone	7	Randomized trials	Serious ^a,b,c^	Serious ^f^	Not serious	Serious ^d^	None	97	92	MD **0.84 nmol/L higher** (− 0.2 lower to 1.9 higher)		⨁◯◯◯ VERY LOW
SHBG	3	Randomized trials	Serious ^a,c^	Serious ^f^	Not serious	Serious ^d^	Publication bias strongly suspected ^e^	31	28	MD − **5.5 nmol/L lower** (− 19.4 lower to 8.2 higher)		⨁◯◯◯ VERY LOW
Cortisol	5	Randomized trials	Serious ^a,c,g^	Very serious ^h^	Not serious	Serious ^d^	None	63	62	MD **17.4 nmol/L lower** (− 88.5 lower to 53.7 higher)		⨁◯◯◯ VERY LOW
IL-6	3	Randomized trials	Serious ^a,c,i^	Serious ^f^	Not serious	Serious ^d^	None	Statistical pooling was not possible for this variable				⨁◯◯◯ VERY LOW
TNF-α	3	Randomized trials	Serious ^a,c,i^	Serious ^f^	Not serious	Serious ^d^	None	Statistical pooling was not possible for this variable				⨁◯◯◯ VERY LOW

CI: Confidence interval; MD: Mean difference

a. Including at least one study with deviations from intended intervention

b. Including at least one study with unclear handling of missing data

c. Including at least one study with an unclear selection of the reported results

d. Small sample size

e. All studies from the same research group

f. Downgraded by 1 due to inconsistency in findings across studies

g. Including at least one study with some concerns with the randomization process

h. Downgraded by 2 due to inconsistency in findings across studies

i. Including at least one study with bias in the measurement of the outcome

Table 5 shows a sensitivity analysis of how pooled exercise training affects hormonal response with and without studies, including prepubertal participants [29, 43, 54, 56, 64]. The withdrawal of the studies with a prepubertal group only impacted the main analysis for GH. The primary main effect for GH was not significant, but when the prepubertal group in the Zakas et al. study [54] was removed from the analysis, a significant main effect was observed (*p* = 0.04). The main analysis of IGF-I, testosterone, SHBG, and cortisol was not affected by the withdrawal of the prepubertal groups.

**Table 5:** Sensitivity analysis per hormonal outcome with all groups or only adolescents (> 12 years old)

Outcome	Study group	Mean difference [95% CI]	Overall effect
GH	All studies (primary main effect)	0.48 ng/mL (− 0.02, 0,99)	*Z* = 1.87, *p* = 0.06
	Only adolescents (> 12 years old)	0.59 ng/mL (0.03, 1.15)	*Z* = 2.05, *p* = 0.04
IGF-I	All studies (primary main effect)	− 22.9 ng/mL (− 47.92, 2.12)	*Z* = 1.79, *p* = 0.07
	Only adolescents (> 12 years old)	− 18.11 ng/mL (− 46.75, 10.54)	*Z* = 1.24, *p* = 0.22
Testosterone	All studies (primary main effect)	0.84 nmol/L (− 0.22, 1.90)	*Z* = 1.54, *p* = 0.12
	Only adolescents (> 12 years old)	1.56 nmol/L (− 0.82, 3.93)	*Z* = 1.28, *p* = 0.20
SHBG	All studies (primary main effect)	− 5.58 nmol/L (− 19.39, 8.23)	*Z* = 0.79, *p* = 0.43
	Only adolescents (> 12 years old)	0.70 nmol/L (− 16.41, 17.81)	*Z* = 0.08, *p* = 0.94
Cortisol	All studies (primary main effect)	− 17.40 nmol/L (− 88.50, 53.70)	*Z* = 0.48, *p* = 0.63
	Only adolescents (> 12 years old)	− 43.32 nmol/L (− 108.5, 21.89)	*Z* = 1.30, *p* = 0.19

### Subgroup Analyses: Effect of Training Type

*Testosterone* The subgroup analysis identified a significant difference in testosterone concentration pre- and post-training between endurance and resistance training (*p* = 0.008, Fig. 4). Testosterone concentration increased significantly after resistance training compared to a control group (mean difference: 3.42 nmol/L [95% CI 0.92 to 5.92], *Z* = 2.68, [*p* = 0.007]) while no difference was observed after endurance training (mean difference: − 0.01 nmol/L [95% CI − 0.41 to 0.39], *Z* = 0.03,[*p* = 0.97]). The between-study heterogeneity for both resistance training (*I*^2^ = 29%, *p* = 0.24) and endurance training (*I*^2^ = 0%, *p* = 0.97) was low.

*SHBG* No subgroup difference was found for SHBG (Fig. 5) concentrations between training types (*p* = 0.59). Resistance training did not reveal any clear effect on SHBG (mean difference: − 10.46 nmol/L [95% CI − 36.69 to 15.76], *Z* = 0.78 [*p* = 0.43]). Only one study was found examining SHBG and endurance training.

*Cortisol:* Further, no difference between training types (Fig. 6) was evident in cortisol pre- to post-training (*p* = 0.49, *I*^2^ = 0%). No difference in cortisol concentration was evident after either resistance training (*p* = 0.81, *Z* = 0.24) or endurance training (*p* = 0.48, *Z* = 0.70).

*GH-IGF-I:* No study analyzing GH and IGF-I and resistance training was found, and therefore subgroup analyses was not conducted. Within-group analysis (Fig. 2 and Fig. 3) showed that endurance training resulted in a small non-significant increase in GH (mean difference: 048 ng/mL; [95% CI − 0.02 to 0.99]; *I*^2^ = 0%, *Z* = 1.87, *p* = 0.06) pre- to post-training while IGF-I decreased following endurance training (mean difference: − 22.90 ng/mL [95% CI − 47.92 to 2.12], *Z* = 1.79 [*p* = 0.07]). A summary of the results is presented in Table 6.

**Table 6:** Summary of changes in resting concentration of hormones in children and adolescents following exercise training

Hormone	Exercise training	Resistance training	Endurance training	Concurrent training
Growth hormone (GH)	↑–	?	↑–	?
IGF-I	↓–	?	↓	?
Testosterone	?	↑	–	?
SHBG	?	?	?	?
Cortisol	?	?	?	?

↑ Increase, ↓ Decrease, ? Unclear, – no change

### Proinflammatory Cytokines

Three studies [43, 66, 67] were found examining the effects of exercise training on IL-6 and TNF-α. The overall (GRADE) certainty of evidence was very low for IL-6 and TNF-α (Table 4). All studies used endurance training as a training mode, and no study was found using resistance training. Since only two of the studies [43, 66] reported quantitative measures of pre- and post-values of the outcomes, the results are only described. Only one study reported changes in IL-6 [66] and TNF-α [43] with exercise training. Rosenbaum et al. [66] examined 13 weeks of aerobic-type training in junior-high-school students (13–14 years old) (Table 2) and found reduced resting concentration of IL-6 but without any change in TNF-α after the training period. Scheett et al. [43] also examined the effects of endurance-type training (5 weeks) in prepubertal and early pubertal students (Table 2). They found a significant increase in TNF-α but no change in IL-6 after the training period. Lastly, Andrade et al. [67] reported no effects of 6 weeks of moderate-intensity (80% HR_max_) endurance training on TNF-α or IL-6 in children (age: 11–12 years).

### Risk of Bias Assessments

All studies were graded according to the Cochrane risk of bias assessments. Overall, the risk of bias scores for the included studies were graded as moderate (Fig. 7). The randomization process revealed concerns for one study [65] due to lack of information. For most studies, the risk of bias due to missing outcome data showed low concerns except for one study [63], which showed some concerns since results were not reported for all participants. Measurements bias of the outcomes showed some concern in one study [66] because of some concerns about whether data collection was different between the groups. Selective reporting of the results raised some concerns in almost all included studies due to the absence of an a priori published study protocol or pre-specified analysis plan. Only one of the included studies [67] reported following an “a priori” published protocol.

## Discussion

In this systematic review with meta-analysis, we examined if exercise training and training type influence chronic changes in resting concentrations of hormones (GH, IGF-I, testosterone, SHBG, cortisol) and proinflammatory cytokines (IL-6 and TNF-α) in healthy children and adolescents (< 18 years). The analysis revealed that exercise training generally seems to have small effects on the resting concentrations of hormones in children and adolescents. Resting levels of GH increased significantly after endurance training in adolescents (MD = 0.59 ng/mL, *p* = 0.04) but not when the analysis included children. GH response to training may depend on maturity, in line with studies on acute training that reported more significant GH response in pubertal than prepubertal children following endurance-type training [5, 32, 33, 36, 40, 41]. Previous research suggested that exercise training has an antagonistic effect on the chronic changes in the GH-IGF-I axis [43, 68]. We confirmed an increase in resting concentrations of GH after exercise in adolescents; however, no significant difference was observed for IGF-I. In addition, proinflammatory cytokines have been suggested to increase after short-term training [22, 43]. We identified few studies investigating exercise training and the effect on resting concentrations of IL-6 and TNF-α, and meta-analyses were therefore not possible. Based on current evidence, data suggest unclear effects of training on cytokines in children and adolescents. Three studies were found [43, 66, 67], and only one study found a significant change in TNF-α [43] and IL-6 [66]. It was not possible to analyze the effects of training types on IL-6 or TNF-α since all studies used endurance training as a training regime.

Furthermore, testosterone generally increased or showed no change in resting concentration following exercise training. Subgroup analysis revealed a significant increase in testosterone concentration after resistance training (MD = 3.42 nmol/L) but not after endurance training (MD = − 0.01 nmol/L). Training type may affect adaptations to exercise training, although this may be specific to some hormones only. It was not possible to examine if training type is important for GH or IGF-I since all studies used endurance training. If exercise training and training type affect children and adolescents' concentration of SHBG and cortisol remains unclear due to limited evidence.

Exercise training positively affects children and adolescents physical and physiological characteristics such as muscular strength, power, and endurance [14, 69–72]. These effects become more notable upon the onset of puberty and due to increases in the concentration of anabolic hormones (e.g., testosterone) [73]. Some evidence suggests that the magnitude of training adaptations following resistance training is maturity-dependent and less effective before the growth spurt [74]. The hormonal adaptations following exercise training are expected to be more significant in pubertal than prepubertal children due to their higher basal hormonal levels [75]. Biological age is a possible confounding factor that should be examined in future analysis. Only a few studies measured or reported biological age in this meta-analysis to conduct a subgroup analysis. The analysis without the prepubertal groups showed no change in the main effect for the selected hormones except GH, which showed a significant main effect when the prepubertal children were excluded.

The study by Zakas et al. [54] was one of the few included studies that directly compared different age groups and concluded that maturation affects the hormonal responses to moderate- to high-intensity interval training in males [54]. More specifically, endurance training elevated chronic resting concentrations of serum testosterone and GH in pubertal (13 yrs) and adolescent (16 yrs) males but not in prepubertal males (10 yrs). The age-related difference in hormonal concentrations should be attributed to differences in the maturity of the examined groups [76]. However, evidence is rather scarce and conflicting. In a study by Tsolakis et al. [55], eight weeks of upper-body moderate-intensity resistance training twice a week showed no significant difference in testosterone concentrations between prepubertal and pubertal males. Tsolakis et al. [55] reported a 124% increase in testosterone pre- to post-training for the prepubertal group (11–13 years), which was higher compared to the 32% increase observed in the pubertal group. The maturation stage in the study was based on external genitals and pubic hair development, but no evaluation of testicle development was reported [55]. It is possible that the prepubertal group in Tsolakis et al. [55] study was a mix of early pubertal and prepubertal, based on the high variability of participants’ anthropometrical characteristics (height: 152 ± 5.9 cm; weight: 43 ± 9.5 kg). To date, no similar study exists on females. It is not clearly understood how maturation affects training-induced hormonal responses, but it seems that aspects like training duration, training type, and which hormone are examined should be taken into account when addressing that question/matter.

On a similar topic, many of the included studies in this systematic review and meta-analysis did not include the maturity status of the examined population [59, 60, 65–67]. This is of particular importance since it is well known that a physiological rise in hormonal levels occurs during puberty, possibly affecting the adaptations to training. In addition, there is a wide range of variability in growth as well as the tempo of maturation between individuals of the same chronological age group [77]. Further, researchers investigating endocrine adaptations to training in pediatric populations mainly examine adolescents [9], which is where the largest variability tends to exist. This is especially true for adolescents around the growth spurt [78–80]. Future research is urged to attempt to control for maturity status.

The literature containing data on female groups (children and adolescents) is substantially smaller than for its male counterparts [81]. Only two studies [26, 61], out of the 14 included in this review, examined females, and both used endurance training as the training type. No eligible study was found examining the effects of resistance training on hormones or cytokines in healthy females, which is a limitation that future research should address. Further, two eligible studies included in the systematic review used a mixed population sample [66, 67], making interpretation difficult. Females enter puberty on average two years earlier than males and have different physiology with a higher level of sex steroids and GH/IGF-I, which may have a different hormonal response to the training process [82]. Currently, there is not enough evidence to meta-analyze sex-specific differences in hormonal response to training. Data from meta-analysis examining strength gains after resistance training have reported a greater effect size in children and adolescent males [83] than females [81]. Sex differences are attributed to females’ lower increase in testosterone [82] along with increases in circulating estrogens that result in lower muscle mass and promote fat distribution [84].

None of the studies included in the meta-analysis examined the effects of concurrent training on hormonal or cytokine responses in healthy children and adolescents. Other studies on overweight and obese children and adolescents have used concurrent training [45, 85] to examine its effects on insulin and glucose. Concurrent training has been gaining interest in exercise science mainly because of the interference effect, stating that the mix of both endurance and resistance training together in the same training session might be less effective than single-mode training [86]. The interference effect has sometimes been evident [86], but research on children and adolescents is scarce. Children using concurrent training do not seem to experience an interference effect as seen in adult studies. In contrast, concurrent training for children and adolescents seems more effective than endurance training or resistance training separately [2]. In a recent systematic review, concurrent training in youth had similar and even better training effects in some selected measures of physical fitness compared to only resistance or endurance training separately [2]. Concurrent training was more effective than resistance training to develop muscular power and more effective than endurance training on developing athletic performance, endurance, and work economy [2]. It has been suggested that the concurrent training-related interference effect is age-dependent and present in adolescents (13–18 yrs.) but not in children (6–12 yrs.) [2]. In adults, acute hormonal response to concurrent training in the same training session can be metabolically demanding, increasing cortisol concentration and potentially suppressing testosterone post-loading [87]. Moreover, the order of exercise also seems to play a role in exercise-induced hormonal responses. Goto et al. [88] showed that endurance exercise conducted before strength training might suppress the hormonal release of GH. A possible explanation might be the accumulation of fatty acids. Others have shown that endurance training conducted before resistance training elevates testosterone concentration but not when resistance training was conducted first [89]. Taken together, there is a knowledge gap in how children and adolescents' hormonal responses adapt to concurrent training.

The main strength of this systematic review and meta-analysis is that we conducted a comprehensive systematic search with broad inclusion of several training-related hormones and cytokines and their response to exercise training and training type in children and adolescents. By summarizing and integrating results from a number of individual, typically small sample-sized studies, we could increase precision in estimating the effects of exercise training on hormones. However, the analysis has some limitations that need to be interpreted with some caution. Only one investigator screened the titles which in best practice is done by two independent investigators. In order to decrease the risk of missing studies, we manually searched all included studies' reference lists but did not find any eligible studies. In general, the meta-analysis had low-to-moderate heterogeneity, similar to other meta-analyses examining hormonal adaptations in children and adolescents [45]. The moderate-to-high risk of bias in the included studies might partially explain the results. The risk of bias is partly difficult to assess in training studies since blinding study participants is not practically feasible, resulting in lower scores [90]. In addition, previous systematic reviews [91, 92] of training studies report that the studies usually are of low to medium quality evidence. Other challenges that we faced during the data analysis were that most of the studies included small sample sizes had an inadequate description of training variables, and their training duration was typically short. The large variation of training protocols within each training type is likely to contribute to the observed heterogeneity in the meta-analysis. Other studies have suggested that both intensity and training volume are likely important confounders when examining training effects on hormones [5]. In this study, we showed that training type (endurance vs. resistance training) has a training-specific impact on some important hormones. It is important to note that training type could not be compared for all outcomes due to a lack of studies (e.g., GH-IGF-I axis only examined following endurance training), which should be addressed in future research. More surprising is the lack of studies using concurrent training as a training type. Concurrent training, in theory, might have the combined effect of both endurance training and resistance training and resemble real-life situations for children’s and adolescents’ weekly training. Since WHO guidelines [1] for physical activity in children and adolescents include endurance and muscular strength training, there is a need to examine concurrent training’s effect on hormones and cytokines. Hence, we suggest future studies to explore the effects of the more complex training type, concurrent training, on hormonal and proinflammatory cytokine responses.

## Conclusions

Based on the overall findings of this systematic review with meta-analysis, we conclude that short-term exercise training has small effects on resting hormonal concentrations in healthy children and adolescents. GH response to training may be affected by maturation since GH increases after training only in adolescents but not in children. Based on our results, the type of exercise training affects exercise-induced hormonal adaptations, at least resting testosterone concentrations. Resistance training increases testosterone concentrations, while endurance training has a limited effect. However, significant limitations exist in the current literature, mainly due to few randomized controlled trials examining pediatric hormonal adaptations to exercise training. Our results demonstrated a low certainty in current evidence for the effects of exercise training on hormonal and cytokine outcomes.

Further high-quality, robust training studies investigating the effects of resistance training, endurance training, and concurrent training on both hormones and cytokines are needed to elucidate training’s specific effects. There is a knowledge gap in pediatric research examining the effects of concurrent training on chronic hormonal response in healthy children and adolescents. From a health perspective, this should be of interest since many public health recommendations for children and adolescents include endurance and resistance training conducted weekly. Future research is urged to continue investigating the effects of exercise training on chronic adaptations in hormones and cytokines by using different training modalities, controlling for maturation, and targeting female populations in both child and adolescent age groups.

## Abbreviations

CI: Confidence intervals
MD: Mean difference
GH: Growth hormone
IGF-I: Insulin-like growth factor 1
SHBG: Sex-hormone binding globulin
IL-6: Interleukin 6
TNF-α: Tumor necrosis factor-alpha
GRADE: The Grading of Recommendations Assessment, Development and Evaluation
ACTH: Adrenocorticotropic hormone
WHO: World Health Organization
PRISMA: Preferred Reporting Items for Systematic Reviews and Meta-Analysis
PICO: Population (P), intervention (I), comparator (C), and outcomes
SD: Standard deviation
HR_max_: Maximal heart rate

## Declarations

### Availability of Data and Materials

Available upon reasonable request.

## References

[1] WHO. Global recommendations on physical activity for health. 2011. 26180873

[2] Gäbler M, Prieske O, Hortobágyi T, Granacher U (2018). The effects of concurrent strength and endurance training on physical fitness and athletic performance in youth: A systematic review and meta-analysis. Front Physiol.

[3] Riddell MC (2008). The endocrine response and substrate utilization during exercise in children and adolescents. J Appl Physiol.

[4] Rubin DA, Tufano JJ, McMurray RG. Endocrinology of physical activity and sport. In Hackney AC, Constantini NW, editors. Cham: Springer; 2020.

[5] Kraemer WJ, Ratamess NA (2005). Hormonal responses and adaptations to resistance exercise and training. Sport Med.

[6] Zouhal H, Jayavel A, Parasuraman K, Hayes L, Tourny C, Rhibi F (2021). Effects of exercise training on anabolic and catabolic hormones with advanced age: a systematic review. Sports Med.

[7] Falk B, Eliakim A (2014). Endocrine response to resistance training in children. Pediatr Exerc Sci.

[8] Legerlotz K, Marzilger R, Bohm S, Arampatzis A (2016). Physiological adaptations following resistance training in youth athletes—A narrative review. Pediatr Exerc Sci.

[9] Boisseau N, Delamarche P (2000). Metabolic and hormonal responses to exercise in children and adolescents. Sport Med.

[10] Löfqvist C, Andersson E, Gelander L, Rosberg S, Blum W, Albertsson WK (2001). Reference values for IGF-I throughout childhood and adolescence: a model that accounts simultaneously for the effect of gender, age, and puberty. J Clin Endocrinol Metab.

[11] Albertsson-Wikland K, Rosberg S (1988). Analyses of 24-hour growth hormone profiles in children: relation to growth. J Clin Endocrinol Metab.

[12] Eliakim A (2016). Endocrine response to exercise and training-closing the gaps. Pediatr Exerc Sci.

[13] Round JM, Jones DA, Honour JW, Nevill AM (1999). Hormonal factors in the development of differences in strength between boys and girls during adolescence: a longitudinal study. Ann Hum Biol.

[14] Behringer M, Vom HA, Matthews M, Mester J (2011). Effects of strength training on motor performance skills in children and adolescents: a meta-analysis. Pediatr Exerc Sci.

[15] Guy JA, Micheli LJ (2001). Strength training for children and adolescents. J Am Acad Orthop Surg.

[16] Malina RM (2006). Weight training in youth-growth, maturation, and safety: An evidence-based review. Clin J Sport Med.

[17] Kanehisa H, Ikegawa S, Tsunoda N, Fukunaga T (1995). Strength and cross-sectional areas of reciprocal muscle: groups in the upper arm and thigh during adolescence. Int J Sports Med.

[18] O’Brien TD, Reeves ND, Baltzopoulos V, Jones DA, Maganaris CN (2010). In vivo measurements of muscle specific tension in adults and children. Exp Physiol.

[19] O’brien TD, Reeves ND, Baltzopoulos V, Jones DA, Maganaris CN (2010). Muscle-tendon structure and dimensions in adults and children. J Anat.

[20] Sekine Y, Hirose N (2021). Maturity-associated variations in resistance exercise-induced hormonal responses in young male athletes. Pediatr Exerc Sci.

[21] Nemet D, Eliakim A (2010). Growth hormone-insulin-like growth factor-1 and inflammatory response to a single exercise bout in children and adolescents. Med Sport Sci.

[22] Nemet D, Oh Y, Kim H-S, Hill M, Cooper DM (2002). Effect of intense exercise on inflammatory cytokines and growth mediators in adolescent boys. Pediatrics.

[23] Nemet D, Rose-Gottron CM, Mills PJ, Cooper DM (2003). Effect of water polo practice on cytokines, growth mediators, and leukocytes in girls. Med Sci Sports Exerc.

[24] Meckel Y, Eliakim A, Seraev M, Zaldivar F, Cooper DM, Sagiv M (2009). The effect of a brief sprint interval exercise on growth factors and inflammatory mediators. J Strength Cond Res.

[25] Le Roith D (2003). The insulin-like growth factor system. Exp Diabesity Res.

[26] LeRoith D, Roberts CT (1993). Insulin-like growth factors and their receptors in normal physiology and pathological states. J Pediatr Endocrinol Metab.

[27] Kelly PJ, Eisman JA, Stuart MC, Pocock NA, Sambrook PN, Gwinn TH (1990). Somatomedin-c, physical fitness, and bone density. J Clin Endocrinol Metab.

[28] Poehlman ET, Copeland KC (1990). Influence of physical activity on insulin-like growth factor-I in healthy younger and older men. J Clin Endocrinol Metab.

[29] Eliakim A, Scheett TP, Newcomb R, Mohan S, Cooper DM (2001). Fitness, training, and the growth hormone→insulin-like growth factor I axis in prepubertal girls1. J Clin Endocrinol Metab.

[30] Eliakim A, Brasel JA, Barstow TJ, Mohan S, Cooper DM (1998). Peak oxygen uptake, muscle volume, and the growth hormone-insulin-like growth factor-I axis in adolescent males. Med Sci Sports Exerc.

[31] Pomerants T, Tillmann V, Karelson K, Jürimäe J, Jürimäe T (2006). Ghrelin response to acute aerobic exercise in boys at different stages of puberty. Horm Metab Res.

[32] Marin G, Domené HM, Barnes KM, Blackwell BJ, Cassorla FG, Cutler GB (1994). The effects of estrogen priming and puberty on the growth hormone response to standardized treadmill exercise and arginine-insulin in normal girls and boys. J Clin Endocrinol Metab.

[33] Oliver SR, Rosa JS, Minh TDC, Pontello AM, Flores RL, Barnett M (2010). Dose-dependent relationship between severity of pediatric obesity and blunting of the growth hormone response to exercise. J Appl Physiol.

[34] Bouix O, Brun JF, Fedou C, Raynaud E, Kerdelhue B, Lenoir V (1994). Plasma β-endorphin, corticotrophin and growth hormone responses to exercise in pubertal and prepubertal children. Horm Metab Res.

[35] Sills IN, Cerny FJ (1983). Responses to continuous and intermittent exercise in healthy and insulin-dependent diabetic children. Med Sci Sports Exerc.

[36] Wirth A, TrÄger E, Scheele K, Mayer D, Diehm K, Reischle K (1978). Cardiopulmonary adjustment and metabolic response to maximal and submaximal physical exercise of boys and girls at different stages of maturity. Eur J Appl Physiol Occup Physiol.

[37] Garlaschi C, Di Natale B, Del Guercio MJ, Caccamo A, Gargantini L, Chiumello G (1975). Effect of physical exercise on secretion of growth hormone, glucagon, and cortisol in obese and diabetic children. Diabetes.

[38] Eliakim A, Nemet D, Zaldivar F, McMurray RG, Culler FL, Galassetti P (2006). Reduced exercise-associated response of the GH-IGF-I axis and catecholamines in obese children and adolescents. J Appl Physiol.

[39] Nemet D, Eliakim A, Mills PJ, Meckal Y, Cooper DM (2009). Immunological and growth mediator response to cross-country training in adolescent females. J Pediatr Endocrinol Metab.

[40] Viru A, Laaneots L, Karelson K, Smirnova T, Viru M (1998). Exercise-induced hormone responses in girls at different stages of sexual maturation. Eur J Appl Physiol Occup Physiol.

[41] Pomerants T, Tillmann V, Karelson K, Jürimäe J, Jürimäe T (2008). Impact of acute exercise on bone turnover and growth hormone/insulin-like growth factor axis in boys. J Sports Med Phys Fitness.

[42] Kraemer W, Marchitelli L, Gordon S, Harman E, Dziados J, Mello R (1990). Hormonal and growth factor responses to heavy resistance exercise protocols. J Appl Physiol.

[43] Scheett TP, Nemet D, Stoppani J, Maresh CM, Newcomb R, Cooper DM (2002). The effect of endurance-type exercise training on growth mediators and inflammatory cytokines in pre-pubertal and early pubertal males. Pediatr Res.

[44] Eliakim A, Nemet D (2010). Exercise training, physical fitness and the growth hormone-insulin-like growth factor-1 axis and cytokine balance. Med Sport Sci.

[45] Marson E, Delevatti R, Prado A, Netto N, Kruel L (2016). Effects of aerobic, resistance, and combined exercise training on insulin resistance markers in overweight or obese children and adolescents: a systematic review and meta-analysis. Prev Med (Baltim).

[46] Pham H, Ng J, Adams E, Rubin DA, Castner DM, Judelson DA (2014). Hormonal and metabolic responses to a resistance exercise protocol in lean children, obese children, and lean adults. Pediatr Exerc Sci.

[47] Liberati A, Altman DG, Tetzlaff J, Mulrow C, Gøtzsche PC, Ioannidis JPA, et al. The PRISMA statement for reporting systematic reviews and meta-analyses of studies that evaluate health care interventions: Explanation and elaboration. PLoS Med. 2009. 10.1371/journal.pmed.1000100 PMC2707010 19621070

[48] Page MJ, Moher D, Bossuyt PM, Boutron I, Hoffmann TC, Mulrow CD, et al. PRISMA 2020 explanation and elaboration: updated guidance and exemplars for reporting systematic reviews. BMJ. 2021. 10.1136/bmj.n160 PMC8005925 33781993

[49] Richardson WS, Wilson MC, Nishikawa J, Hayward RS (1995). The well-built clinical question: a key to evidence-based decisions. ACP J Club.

[50] Ryan R, Hill S. Supporting implementation of Cochrane methods in complex communication reviews: Resources developed and lessons learned for editorial practice and policy. Heal Res Policy Syst. 2019. 1–11. 10.1186/s12961-019-0435-0 PMC6437949 30922338

[51] Drevon D, Fursa S, Malcolm A (2017). Intercoder reliability and validity of WebPlotDigitizer in extracting graphed data. Behav Modif Behav Modif.

[52] Atkins D, Best D, Briss P, Eccles M, Falck-Ytter Y, Flottorp S (2004). Grading quality of evidence and strength of recommendations. BMJ.

[53] Broek JL, Akl EA, Alonso-Coello P, Lang D, Jaeschke R, Williams JW (2009). Grading quality of evidence and strength of recommendations in clinical practice guidelines: Part 1 of 3. An overview of the GRADE approach and grading quality of evidence about interventions. Allergy Eur J Allergy Clin Immunol.

[54] Zakas A, Mandroukas K, Karamouzis G, Panagiotopoulou G (1994). Physical training, growth hormone and testosterone levels and blood pressure in prepubertal, pubertal and adolescent boys. Scand J Med Sci Sports.

[55] Tsolakis C, Messinis D, Stergioulas A, Dessypris A (2000). Hormonal responses after strength training and detraining in prepubertal and pubertal boys. J Strength Cond Res.

[56] Tsolakis C, Vagenas G, Dessypris A (2004). Strength adaptations and hormonal responses to resistance training and detraining in preadolescent males. J strength Cond Res.

[57] Higgins JPT, Thompson SG, Deeks JJ, Altman DG (2003). Measuring inconsistency in meta-analyses. Br Med J.

[58] Higgins JPT, Thomas J, Chandler J, Cumpston M, Li T, Page MJ WV. Cochrane Handbook for Systematic Reviews of Interventions version 6.0 (updated July 2019). Cochrane, 2019. Cochrane. 2019.

[59] Gorostiaga EM, Izquierdo M, Ruesta M, Iribarren J, Gonzalez-Badillo JJ, Ibáñez J (2004). Strength training effects on physical performance and serum hormones in young soccer players. Eur J Appl Physiol.

[60] Büyükyazi G, Karamizrak SO, Islegen Ç (2003). Effects of continuous and interval running training on serum growth and cortisol hormones in junior male basketball players. Acta Physiol Hung Acta Physiol Hung.

[61] Gorostiaga EM, Izquierdo M, Iturralde P, Ruesta M, Ibáñez J (1999). Effects of heavy resistance training on maximal and explosive force production, endurance and serum hormones in adolescent handball players. Eur J Appl Physiol Occup Physiol.

[62] Eliakim A, Brasel JA, Mohan S, Barstow TJ, Berman N, Cooper DM (1996). Physical fitness, endurance training, and the growth hormone-insulin-like growth factor I system in adolescent females. J Clin Endocrinol Metab.

[63] Eliakim A, Brasel JA, Mohan S, Wong WL, Cooper DM (1998). Increased physical activity and the growth hormone-IGF-I axis in adolescent males. Am J Physiol.

[64] Messinis D, Tsolakis CK, Stergioulas A, Dessypris A (2000). Androgen responses following a two-month endurance training program and one month detraining in prepubertal boys. New Zeal J Sport Med.

[65] Sarabia JM, Fernandez-Fernandez J, Juan-Recio C, Hernández-Davó H, Urbán T, Moya M (2015). Mechanical, hormonal and psychological effects of a non-failure short-term strength training program in young tennis players. J Hum Kinet.

[66] Rosenbaum M, Nonas C, Weil R, Horlick M, Fennoy I, Vargas I (2007). School-based intervention acutely improves insulin sensitivity and decreases inflammatory markers and body fatness in junior high school students. J Clin Endocrinol Metab.

[67] De ALB, Britto MCA, Lucena-Silva N, Gomes RG, Figueroa JN (2014). The efficacy of aerobic training in improving the inflammatory component of asthmatic children. Randomized trial. Respir Med.

[68] Scheett T, Mills P, Ziegler M, Stoppani J, Cooper D (1999). Effect of exercise on cytokines and growth mediators in prepubertal children. Pediatr Res.

[69] Peitz M, Behringer M, Granacher U (2018). A systematic review on the effects of resistance and plyometric training on physical fitness in youth- What do comparative studies tell us?. PLoS ONE.

[70] Lesinski M, Prieske O, Granacher U (2016). Effects and dose-response relationships of resistance training on physical performance in youth athletes: a systematic review and meta-analysis. Br J Sports Med.

[71] Alves AR, Marta C, Neiva HP, Izquierdo M, Marques MC (2018). Concurrent training in prepubertal children: an update. J Hum Sport Exerc.

[72] Baquet G, Van Praagh E, Berthoin S (2003). Endurance training and aerobic fitness in young people. Sport Med.

[73] Viru A, Loko J, Harro M, Volver A, Laaneots L, Viru M (2006). Critical periods in the development of performance capacity during childhood and adolescence. Eur J Phys Educ.

[74] Meylan CMP, Cronin JB, Oliver JL, Hopkins WG, Contreras B (2014). The effect of maturation on adaptations to strength training and detraining in 11–15-year-olds. Scand J Med Sci Sport.

[75] Ankarberg-Lindgren C, Norjavaara E (2004). Changes of diurnal rhythm and levels of total and free testosterone secretion from pre to late puberty in boys: testis size of 3 ml is a transition stage to puberty. Eur J Endocrinol.

[76] Rowland TW, Unnithan VB, MacFarlane NG, Gibson NG, Paton JY (1994). Clinical manifestations of the ‘Athlete’s Heart’ in prepubertal male runners. Int J Sports Med.

[77] Mirwald R, Baxter-Jones ADG, Bailey DA, Beunen G (2002). An assessment of maturity from anthropometric measurements. Med Sci Sports Exerc.

[78] Iuliano-Burns S, Mirwald R, Bailey D (2001). Timing and magnitude of peak height velocity and peak tissue velocities for early, average, and late maturing boys and girls. Am J Hum Biol.

[79] Marshall WA, Tanner JM (1970). Variations in the pattern of pubertal changes in boys. Arch Dis Child.

[80] Marshall WA, Tanner JM (1969). Variations in pattern of pubertal changes in girls. Arch Dis Child.

[81] Moran J, Sandercock G, Ramirez-Campillo R, Clark CCT, Fernandes JFT, Drury B (2018). A meta-analysis of resistance training in female youth: its effect on muscular strength, and shortcomings in the literature. Sports Med.

[82] Tønnessen E, Svendsen IS, Olsen IC, Guttormsen A, Haugen T (2015). Performance development in adolescent track and field athletes according to age, sex and sport discipline. PLoS ONE.

[83] Moran J, Sandercock GRH, Ramírez-Campillo R, Meylan C, Collison J, Parry DA (2017). A meta-analysis of maturation-related variation in adolescent boy athletes’ adaptations to short-term resistance training. J Sports Sci.

[84] Tipton K (2001). Gender differences in protein metabolism. Curr Opin Clin Nutr Metab Care.

[85] García-Hermoso A, Ramírez-Vélez R, Ramírez-Campillo R, Peterson MD, Martínez-Vizcaíno V. Concurrent aerobic plus resistance exercise versus aerobic exercise alone to improve health outcomes in paediatric obesity: a systematic review and meta-analysis. Br. J. Sports Med. 2018. 161–6. 10.1136/bjsports-2016-096605 27986760

[86] Docherty D, Sporer B (2000). A proposed model for examining the interference phenomenon between concurrent aerobic and strength training. Sport Med.

[87] Brownlee K, Moore A, Hackney A (2005). Relationship between circulating cortisol and testosterone: influence of physical exercise. J Sports Sci Med.

[88] Goto K, Higashiyama M, Ishii N, Takamatsu K (2005). Prior endurance exercise attenuates growth hormone response to subsequent resistance exercise. Eur J Appl Physiol.

[89] Rosa C, Vilaça-Alves J, Fernandes H, Saavedra F, Pinto R, dos Reis V (2015). Order effects of combined strength and endurance training on testosterone, cortisol, growth hormone, and IGF-1 binding protein 3 in concurrently trained men. J Strength Cond Res.

[90] Bolger R, Lyons M, Harrison AJ, Kenny IC (2015). Sprinting performance and resistance-based training interventions: a systematic review. J Strength Cond Res.

[91] Bedoya A, Miltenberger M, Lopez R (2015). Plyometric training effects on athletic performance in youth soccer athletes: a systematic review. J Strength Cond Res.

[92] Johnson B, Salzberg C, Stevenson D (2011). A systematic review: plyometric training programs for young children. J Strength Cond Res.

